# Design and Analytic Methods to Evaluate Multilevel Interventions to Reduce Health Disparities: Rigorous Methods Are Available

**DOI:** 10.1007/s11121-024-01676-9

**Published:** 2024-07-01

**Authors:** David M. Murray, Melody S. Goodman

**Affiliations:** 1 Office of Disease Prevention, National Institutes of Health, Bethesda, MD USA; 2 School of Global Public Health, New York University, New York City, NY USA

## Abstract

In June 2022, the NIH Office of Disease Prevention (ODP) issued a Call for Papers for a Supplemental Issue to Prevention Science on Design and Analytic Methods to Evaluate Multilevel Interventions to Reduce Health Disparities. ODP sought to bring together current thinking and new ideas about design and analytic methods for studies aimed at reducing health disparities, including strategies for balancing methodological rigor with design feasibility, acceptability, and ethical considerations. ODP was particularly interested in papers on design and analytic methods for parallel group- or cluster-randomized trials (GRTs), stepped-wedge GRTs, group-level regression discontinuity trials, and other methods appropriate for evaluating multilevel interventions. In this issue, we include 12 papers that report new methods, provide examples of strong applications of existing methods, or provide guidance on developing multilevel interventions to reduce health disparities. These papers provide examples showing that rigorous methods are available for the design and analysis of multilevel interventions to reduce health disparities.

We are pleased to present this Supplemental Issue to Prevention Science on Design and Analytic Methods to Evaluate Multilevel Interventions to Reduce Health Disparities. The National Institutes of Health (NIH) Office of Disease Prevention (ODP) recognized in 2021 that NIH would be investing increasingly in research to evaluate these interventions. As a result, ODP sought to develop a Supplemental Issue that would provide new methods for that research as well as guidance and strong examples for existing methods.

One of the views often expressed in discussions involving design and analytic methods for multilevel interventions to reduce health disparities is that it is impossible to use rigorous clinical trial methods for community-based research in marginalized, minoritized, or underserved populations. The purpose of the commentary is to disagree with that perspective: we point to the 12 papers in this Supplemental Issue of Prevention Science as evidence to the contrary.

## Papers Focused on New Methods

SWGRTs have become increasingly popular since Hussey and Hughes published the first methods paper on this design in 2007 (Hussey & Hughes, 2007). The standard methods presented in that paper assume the intervention effect is rapid and sustained. More recently, Kenny et al. and Maleyeff et al. showed that standard methods can give very misleading estimates for intervention effects and standard errors if the intervention effect varies over time (Kenny et al., 2022; Maleyeff et al., 2023). Such a pattern is more likely if the intervention lasts more than a few months and if there are multiple follow-up measurements. Kenny et al. and Maleyeff et al. provided analysis and sample size estimation guidance to address time-varying intervention effects in cross-sectional SWGRT designs (Kenny et al., 2022; Maleyeff et al., 2023). Hughes et al., in this Supplemental Issue, extend Kenny et al. (2022) to cohort SWGRT designs using the example of a trial to evaluate a multilevel intervention to address health disparities in blood pressure control (Hughes et al., 2023). The methods of Kenny et al. and Hughes et al. for addressing time-varying intervention effects have already been incorporated into the sample size calculator on the NIH Research Methods Resources website (https://researchmethodsresources.nih.gov/Tools#swgrt).

Parallel GRTs have been increasing in use since the early 1990s, and there are now hundreds of methods papers and several textbook treatments for that design (Campbell & Walters, 2014; Donner & Klar, 2000; Eldridge & Kerry, 2012; Hayes & Moulton, 2017; Murray, 1998). Wang et al., in this Supplemental Issue, present methods for sample size calculation for tests of subgroup-specific intervention effects in the context of a parallel GRT (Wang et al., 2023). Methods have been available for sample size calculation for the difference in subgroup-specific effects (e.g., Murray, 1998) but not for an intervention effect within a single subgroup. Wang et al. show that the power for a single subgroup-specific effect is generally better than for the difference between two subgroup-specific effects.

Previous studies employing a parallel GRT or a SWGRT design randomize only groups or clusters to study arms. Sperger et al., in this Supplemental Issue, describe a multilevel intervention stepped wedge design (MLI-SWD) that combines a group- or cluster-level intervention with an individual-level intervention and describe analytic and sample size methods for the evaluation of their individual effects as well as their joint effect (Sperger et al., 2024). Their methods are quite flexible and could accommodate cross-sectional and cohort designs, situations where all participants begin as members of a cluster, and other situations where participants join their cluster after baseline measurement and after the individual-level intervention has begun. They illustrate their methods in a hypothetical study to evaluate an intervention to improve diabetes-related outcomes in small towns and rural areas. They note that additional work is needed to refine their methods to accommodate time-varying intervention effects (Hughes et al., 2023; Kenny et al., 2022; Maleyeff et al., 2023).

The Multiphase Optimization Strategy (MOST) is an excellent strategy for investigators who seek to maximize the strength of their multilevel intervention (Collins, 2018; Collins & Kugler, 2018; Collins et al., 2021). In this Supplemental Issue, Strayhorn et al. extend the methods for MOST to allow investigators to optimize for health equity (Strayhorn et al., 2024). In a hypothetical case study with simulated data, they show how this extended version of MOST can be applied. They also show how the structure of an optimized intervention can vary when it is optimized for health equity compared to when it is optimized for other criteria.

Investigators do not always consider potential unintended consequences when choosing outcome measures to evaluate their multilevel intervention. Guastaferro et al., in this Supplemental Issue, describe a simulation approach to selecting outcome measures that allow investigators to consider the potential consequences of different methods of operationalizing their outcomes (Guastaferro et al., 2023). Some methods may reduce a health disparity while others may increase that disparity, and those effects may vary across population segments. Considering the factors identified in this paper can allow investigators to operationalize an outcome to avoid unintended consequences for equity.

Most trials to evaluate interventions to address health disparities focus on the primary outcome, usually measured in participants. Jackson et al., in this Supplemental Issue, describe an analytic approach that estimates total effects for the entire sample and for the treated sample and direct effects that are appropriate for decision-based outcomes that may be measured in providers (Jackson et al., 2024). Their total effect is the intention-to-treat effect and represents the total effect of the intervention on disparity for the primary outcome. Their direct effect is the effect of the intervention on disparity for decision-based outcomes. Importantly, the two effects are estimated with regression adjustment for different sets of covariates. They describe a simulation-based approach to sample size estimation and illustrate their methods using a multilevel healthcare intervention to reduce racial and ethnic disparities in hypertension control.

Treatment effect heterogeneity is increasingly of interest in group- or cluster-randomized trials for both parallel and stepped wedge designs. Williamson et al. describe methods to evaluate treatment effect heterogeneity in a parallel group- or cluster-randomized trial when a group- or cluster-level outcome is used in the analysis (Williamson et al., 2023). They report that sufficient power is available for such heterogeneity only for individual-level variables in individual-level models. If outcomes are defined at the group or cluster level, the power to detect heterogeneity of treatment effects is much more limited. They illustrate this issue in a trial evaluating the effect of an intervention on increasing COVID-19 booster vaccination rates at long-term care centers.

One of the common situations that methodologists face, particularly when working with a new team of collaborators, is to work through basic questions that will affect power and dictate the design and analytic plan for the trial. Harrall et al., in this Supplemental Issue, review methods for addressing three of the most important questions: how to choose the unit of randomization, how to choose the primary outcome, and how to approach subgroup analysis for a parallel GRT (Harrall et al., 2024). Their discussion focuses on optimizing power and reducing sample size and cost and is presented in the context of a trial to evaluate a telehealth vs in-person intervention to reduce cardiovascular risk factors.

## Strong Examples of the Application of Existing Methods

In addition to the papers that present new methods, several papers in this Supplemental Issue provide strong examples of the application of existing methods. For example, Guilamo-Ramos et al. (2024) describe a parallel GRT to evaluate the Nurse-Community-Family Partnership intervention in public housing in the South Bronx, New York. Households were randomized to study arms in a 2:1 ratio, intervention to control. The intervention was delivered over five months. Data were collected at baseline, monthly during months 1–6, and again at nine months from all consented household members ten or older. Data will be analyzed using a random-coefficients model, which has been shown to protect the type 1 error rate under conditions common in GRTs (Moyer et al., 2022; Murray et al., 1998). Power was based on the planned analytic model and reflected realistic estimates of the complex correlation structure expected from the design.

Most trials compare an intervention to a usual care arm. Houghton et al., in this Supplemental Issue, take a different approach in which both arms receive the intervention but differ in the method for intervention delivery. They describe a parallel GRT with a staggered start to randomize 30 housing units to a one-year multilevel intervention to increase access to healthy foods and sexual health care or to a control arm that includes many of the intervention components delivered in a different way (Houghton & Adkins-Jackson, 2024). As a result, the comparison is focused on the method of delivery. They evaluate the intervention using mixed models to account for the clustering of participants within housing units.

Conducting research in indigenous populations presents special challenges and requires special methods. Rink et al. (2024) present three case studies that describe their approach to designing, implementing, and evaluating a multilevel intervention to reduce health disparities in an American Indian/Alaska Native population. In their evaluation case study, they describe a small and relatively inexpensive SWGRT that was cleverly designed to maximize power given only five clusters and four sequences by concentrating data collection immediately before, during, and after the delivery of the intervention. They avoided the problems recently identified for time-varying intervention effects in a stepped wedge design (Hughes et al., 2023; Kenny et al., 2022; Maleyeff et al., 2023) by limiting data collection to a single follow-up measure. This paper provides a good example of how a rigorous evaluation can be conducted for a multilevel intervention to reduce health disparities without requiring a large study.

Most GRTs and SWGRTs evaluate one intervention in a two-arm trial. Mulawa et al. provide an example of how to evaluate a hypothetical school-based multilevel intervention to promote mental health equity using an anti-racist approach to intervene at three levels: macro (school system), meso (school), and micro (family and student) (Mulawa et al., 2024). In the first stage, all schools receive the system-level intervention, evaluated in a pre-post design. The following year, schools will be randomized to receive the meso-level intervention. Within each cluster, families will be randomized to receive the micro intervention. They describe their hypothetical example’s design, sample size issues, and analytic methods.

## Guidance for Intervention Development

Several papers in the Supplemental Issue also offer guidance on developing multilevel interventions to reduce health disparities. For example, Guilamo-Ramos et al. (2024) present a heuristic framework for multilevel structural determinants of health (SDOH) intervention research that guided the development of their Nurse-Community-Family Partnership intervention. Houghton & Adkins-Jackson (2024) use critical race theory and intersectionality to construct a structural intervention to improve menstrual cycle health among persons living in food and healthcare deserts in Northern Manhattan. Rink et al. (2024) use two of their case studies to describe the process they recommend for collaboration among multiple, diverse tribal partners and academic investigators to develop a multilevel intervention to address health disparities in American Indian/Alaska Native communities and to develop culturally appropriate methods to implement that intervention.

## The NIH Research Methods Resources Website

We close by pointing readers to the NIH Research Methods Resources (RMR) website (https://researchmethodsresources.nih.gov/). This site provides guidance for investigators planning a clinical trial to evaluate an intervention. Though not focused exclusively on multilevel interventions to reduce health disparities, most of the methods presented on the RMR website are applicable to such interventions.

The RMR website focuses on parallel group- or cluster-randomized trials (GRTs), individually randomized group treatment (IRGT) trials, stepped wedge group- or cluster-randomized trials (SWGRTs), and group- or cluster-based regression discontinuity designs (RDDs). The RMR website provides background, key references, and a sample size calculator for each of these designs. We consider the RMR material as important background that will help readers appreciate the papers included in this Supplemental Issue. Those papers build on the methods presented on the RMR website, and, as noted above, NIH has already incorporated some of the new methods reported in the Supplemental Issue into the material on the RMR website.

## Summary

Taken together, this collection of papers makes clear that rigorous clinical trial methods can be applied to evaluate interventions to reduce health disparities. Many of the papers provide new design, analytic, and sample size methods for GRTs and SWGRTs. Others provide strong examples of applying existing methods for GRTs and SWGRTs. Several offer guidance on intervention development for trials to address health disparities. Randomization played a central role in each paper, contrary to the perception by some that randomized trials are not ethical or even possible in studies conducted in marginalized, minoritized, or underserved populations. In one case, only five groups or clusters were required (Rink et al., 2024), demonstrating that large studies are not always necessary in a randomized trial of a community-based intervention.

Notably, all papers considered for this Supplemental Issue used methods for parallel GRTs or SWGRTs. None used methods for the more traditional randomized controlled trial (RCT) or individually randomized group treatment (IRGT) trials. This is consistent with the focus on evaluating multilevel interventions, where hierarchical designs are the natural approach.
